# Effect of body mass index on clinical outcomes after robotic cardiac surgery: is there an obesity paradox?

**DOI:** 10.1186/s12872-023-03277-w

**Published:** 2023-05-23

**Authors:** Wenjun Wu, Renzhong Ding, Jianming Chen, Ye Yuan, Yi Song, Manrong Yan, Yijie Hu

**Affiliations:** Department of Cardiovascular Surgery, Daping Hospital, Army Medical University, No.10 Changjiang Branch Road, Yuzhong District, Chongqing, 400042 P.R. China

**Keywords:** Robotic surgery, Cardiac surgery, Cardiopulmonary bypass, Body mass index, Clinical outcome, Acute kidney injury, Obesity paradox

## Abstract

**Background:**

To investigate the effect of body mass index (BMI) on clinical outcomes after robotic cardiac surgery, and to explore the postoperative obesity paradox.

**Methods:**

The data of 146 patients who underwent robotic cardiac surgery under cardiopulmonary bypass (CPB) from July 2016 to June 2022 in Daping Hospital of Army Medical University were retrospectively analyzed, and their demographic data and related clinical data were statistically analyzed. The mean age was (42.88 ± 13.01) years, 55 (37.67%) were male and 91 (62.33%) were female. Patients were divided into 3 groups according to preoperative BMI: lean group (BMI < 18.5 kg/m^2^; *n* = 17; 11.64%), normal group (BMI 18.5 kg/m^2^ to 23.9 kg/m^2^; *n* = 81; 55.48%), and overweight and obese group (BMI ≥ 24 kg/m^2^; *n* = 48; 32.88%). Multivariate analysis was performed to compare clinical outcomes across BMI groups.

**Results:**

Preoperative data in different BMI groups showed that there were statistically significant differences in age, height, weight, body surface area (BSA), diabetes, left atrial anteroposterior diameter (LAD), triglyceride (TG), and high-density lipoprotein (HDL) (all *P* < 0.05). Postoperative clinical outcomes showed that there was no statistical difference between the lean group and the normal group; the intensive care unit stay and postoperative hospital stay in the overweight and obese group were significantly higher than those in the normal group (*P* < 0.05), and the risk of postoperative cardiac surgery-related acute kidney injury (CSA-AKI) was significantly increased (*P* = 0.021); further Multiple Binary Logistic Regression Analysis suggested that preoperative TG (*OR* = 1.772, 95% *CI* 1.068–2.942, *P* = 0.027) and operation time ≥ 300 min (*OR* = 3.823, 95% *CI* 1.098–13.308, *P* = 0.035) were independent risk factors for postoperative CSA-AKI.

**Conclusions:**

Overweight and obese patients had significantly prolonged intensive care unit stay and postoperative hospital stay after robotic cardiac surgery, and significantly increased incidence of postoperative CSA-AKI, which did not support the obesity paradox; preoperative TG and operation time ≥ 300 min were independent risk factors for postoperative CSA-AKI.

**Supplementary Information:**

The online version contains supplementary material available at 10.1186/s12872-023-03277-w.

## Background

Obesity is generally considered to be an important risk factor for cardiovascular death, leading to increased morbidity, mortality, and health care costs [1, 2], but studies have found that obesity is associated with reduced mortality after cardiac surgery and has a better prognosis, that is the obesity paradox [3, 4]. In recent years, several studies have suggested that obese patients may be at no different risk for cardiac surgery than non-obese patients, particularly in terms of mortality [5–7]. However, these studies are limited by single-center, small-sample studies, disease types. Furthermore, it remains unclear whether there is an obesity paradox in patients following robotic cardiac surgery. The aim of this study was to investigate the effect of body mass index (BMI) on early clinical outcomes after robotic cardiac surgery under cardiopulmonary bypass (CPB) and to investigate the postoperative obesity paradox.

## Patients and methods

### Patients

One hundred and forty-six patients who underwent cardiac surgery under CPB from July 2016 to June 2022 in Daping Hospital of Army Medical University were selected. Inclusion criteria: (1) Aged ≥ 18 years old, gender, race not limited; (2) Preoperative vascular assessment to determine the suitability for surgery, including: thoracic and abdominal computed tomography (CT), transthoracic echocardiography (TTE), transesophageal echocardiography (TEE), lower limb vascular ultrasonography; (3) Our department performed robotic cardiac surgery, including simple or combined: mitral valve replacement, mitral valve plasty, aortic valve replacement, tricuspid valve plasty, tricuspid valve replacement, congenital heart disease correction, cardiac tumor resection; (4) Patients with complete electronic medical records, especially perioperative data. Exclusion criteria: (1) Age < 18 years, emergency surgery, chronic kidney disease, arrhythmia; (2) non-CPB robotic cardiac surgery, such as coronary artery bypass grafting, pericardial cyst resection, etc.; 2. Patients with severe coronary artery disease, aortic calcification, thoracic tissue adhesion, macroangiopathy, iliofemoral artery disease, severe cardiac insufficiency, respiratory insufficiency, liver and kidney dysfunction, coagulopathy; (3) Patients without complete clinical data to be collected. Patients were divided into 3 groups according to preoperative BMI levels: lean group (BMI < 18.5 kg/m^2^; *n* = 17; 11.64%), normal group (BMI 18.5 kg/m^2^ to 23.9 kg/m^2^; *n* = 81; 55.48%), and overweight and obese group (BMI ≥ 24 kg/m^2^; *n* = 48; 32.88%).The study was reviewed and approved by the Ethics Committee of Daping Hospital of Army Medical University (approval number: 2022–276). Informed consent was obtained from all subjects.All methods were carried out in accordance with the ethical standards in the Declaration of Helsinki.

### Data collection

The electronic case system, medical order system and surgical anesthesia system of our hospital were used to collect the patient information. Basic information: hospitalization number, gender, age, height, weight, BMI, body surface area (BSA), obesity classification, hypertension, diabetes, smoking, drinking, New York Heart Association (NYHA) functional classification, left ventricular ejection fraction (LVEF), left ventricular shortening fraction (LVFS), atrioventricular diameter, tricuspid valve pressure gradient, pulmonary arterial pressure (SPAP); perioperative data: operation time, CPB time, aortic cross-clamp time, intraoperative blood product input, drainage volume on the first day after operation, postoperative tracheal intubation time, postoperative intensive care unit stay, postoperative hospital stay, postoperative complications, type of cardiac surgery, peripheral cardiopulmonary bypass intubation; laboratory tests: preoperative and postoperative biochemical indicators and blood routine. According to clinical needs, the criteria for the diagnosis and staging of postoperative cardiac surgery-associated acute kidney injury (CSA-AKI) were: serum creatinine (SCr) increased by ≥ 26.4µmol/L within 24 h, or increased by ≥ 50% compared with the baseline value (the most recent SCr value before surgery), or urine volume < 0.5 ml/(kg·h) for 6 h [8, 9]. In this study, the urine volume data of patients after surgery were not statistically complete, so urine volume was not used to assess renal function. Indications for continuous renal replacement therapy (CRRT) [10] include volume overload, severe metabolic acidosis, metabolite accumulation, hyperkalemia, and low cardiac output syndrome.

### Surgical methods

After successful general anesthesia and tracheal intubation with a double-lumen tube, an esophageal ultrasound probe was placed transesophagically. The patient was placed in a supine position with a right padding height of 30 ℃, abduction of the right upper arm, and low head and feet. The robotic endoscope orifice is located at 6 cm of the fourth intercostal space and anterior axillary line of the right chest wall, avoiding the breast tissue, with a diameter of about 1 cm; the working orifice is located at 3 cm below the fourth intercostal space and endoscopic orifice of the right chest wall, with a diameter of about 2 cm; the left and right robotic arms are located at 4 cm of the second intercostal space, anterior axillary line and 2 cm of the sixth intercostal space and anterior axillary line of the right chest wall, with a diameter of about 1 cm; the active aortic orifice is located at 3 intercostal spaces of the midaxillary line, with a diameter of about 0.5 cm. Peripheral extracorporeal circulation was established by catheterization of the right internal jugular vein, femoral artery and vein of the right lower limb. The surgeon completed the surgical operation before the control table, and assisted the surgeon on the assistant table. Thorough hemostasis was achieved at the end of surgery, and a chest drainage tube was placed in the right operation hole.

### Statistical analysis

Continuous variables with normal distribution were expressed as mean ± standard deviation (SD), or median (interquartile range) when the normal distribution was not confrmed. The comparison of normally distributed variables between two groups was performed using independent-sample t-test. The comparison of non-normally distributed variables was performed using Mann–Whitney *U*-test. Comparisons among three or more groups of continuous variables were analyzed using analysis of variance (One-Way ANOVA, non-normally distributed variables were log transformed). *X*^2^ or Fisher’s exact test was used for categorical data comparisons. Risk factors of postoperative CSA-AKI were analyzed by One-Way ANOVA, then Multiple Binary Logistic Regression Analysis was performed for factors with univariate *P* < 0.01. *P* < 0.05 was considered statistically signifcant. The statistical analysis was conducted using the SPSS 22.0 software.

## Results

### Patient characteristics

According to the inclusion criteria and exclusion criteria, 146 patients who met the study requirements were screened for clinical information, including 55 males and 91 females, with mean age of (42.88 ± 13.01) years, minimum age of 18 years and maximum age of 72 years, mean height of (160.97 ± 7.72) cm, mean weight of (58.33 ± 11.43) kg and mean body surface area of (1.58 ± 0.18) m^2^.(Table 1). Types of operation: 69 cases (47.26%) of atrial septal defect repair, 23 cases (15.75%) of mitral valve replacement, 17 cases (11.64%) of left atrial myxoma resection, 15 cases (10.27%) of mitral valve plasty, 10 cases (6.85%) of ventricular septal defect repair, 4 cases (2.74%) of aortic valve replacement, 3 cases (2.05%) of congenital endocardial cushion defect correction, 2 cases (1.37%) of left ventricular tumor resection, 2 cases (1.37%) of tricuspid valve replacement, 1 case (0.68%) of anomalous pulmonary venous drainage correction, and 20 cases of tricuspid valve plasty at the same time.(Table 2).

**Table 1:** Patient preoperative data

Variable	Normal	Lean	Overweight and 0bese	*P*-value
	(*n* = 81)	(*n* = 17)	(*n* = 48)	
Age, years	42.83 ± 13.07	27.94 ± 9.78^†^	48.25 ± 9.42^†^	<0.001
Male, *n*(%)	24(29.63)	8(47.06)	23(47.92)^†^	0.082
Height, cm	159.44 ± 6.21	163.59 ± 9.17^†^	162.63 ± 8.96^†^	0.025
Weight, kg	53.94 ± 4.78	45.06 ± 5.17^†^	70.44 ± 10.39^†^	<0.001
BSA, m^2^	1.51 ± 0.09	1.42 ± 0.12^†^	1.74 ± 0.18^†^	<0.001
Diabetes, *n*(%)	0(0)	0(0)	3(6.25)^†^	0.044
Hypertension, *n*(%)	9(11.11)	1(5.88)	8(16.67)	0.455
Smoking, *n*(%)	16(19.75)	3(17.65)	9(18.75)	0.976
Alcohol consumption, *n*(%)	11(13.58)	2(11.76)	14(29.17)^†^	0.066
NYHA				
I, *n*(%)	2(2.47)	0(0)	0(0)	0.449
II, *n*(%)	56(69.13)	13(76.47)	26(54.17)^†^	0.132
III, *n*(%)	23(28.39)	4(23.53)	22(45.83)^†^	0.084
LVFS, %	36.64 ± 5.12	34.82 ± 2.04	36.92 ± 4.45	0.267
LVEF, %	66.16 ± 7.31	64.94 ± 2.11	67.04 ± 4.99	0.465
LAD, mm	34.51 ± 7.08	32.29 ± 4.71	37.01 ± 6.72^†^	0.022
LVDs, mm	42.68 ± 7.06	40.53 ± 6.51	43.92 ± 6.68	0.211
RAD, mm	37.79 ± 7.36	38.01 ± 9.51	37.27 ± 7.47	0.914
RVDs, mm	27.37 ± 8.25	29.35 ± 10.86	27.51 ± 7.47	0.666
Tricuspid valve gradient, mmHg	28.25 ± 13.68	24.18 ± 9.34	25.94 ± 9.96	0.344
SPAP, mmHg	37.56 ± 16.91	32.82 ± 11.52	34.02 ± 11.98	0.292
TC, mmol/L	3.96 ± 0.91	3.44 ± 0.83^†^	4.07 ± 1.08	0.062
TG, mmol/L	1.08 ± 0.61	1.11 ± 1.04	1.64 ± 1.01^†^	0.001
LDL, mmol/L	2.38 ± 0.61	2.08 ± 0.54	2.52 ± 0.72	0.058
HDL, mmol/L	1.32 ± 0.46	1.09 ± 0.28	1.13 ± 0.25^†^	0.008
ALT, U/L	20.71 ± 14.77	16.47 ± 7.85	22.26 ± 12.33	0.312
AST, U/L	23.93 ± 9.55	21.91 ± 6.33	21.28 ± 6.88	0.206
TBIL, µmol/L	13.94 ± 7.11	14.84 ± 8.78	14.18 ± 6.11	0.889
DBIL, µmol/L	2.87 ± 2.14	3.14 ± 2.09	2.58 ± 1.28	0.515
ALB, g/L	41.39 ± 4.53	42.18 ± 4.05	41.12 ± 3.29	0.662
PA, mg/L	222.73 ± 47.72	219.31 ± 43.82	235.89 ± 53.33	0.278
SCr, µmol/L	59.84 ± 11.07	62.62 ± 14.06	65.66 ± 18.61^†^	0.085
GFR, %	147.09 ± 39.65	157.91 ± 39.37	135.06 ± 31.25	0.060
UA, µmol/L	316.83 ± 71.67	331.52 ± 75.52	342.62 ± 115.47	0.277
HGB, g/L	136.63 ± 25.16	141.29 ± 13.96	135.08 ± 23.81	0.651
PLT, 10^9^/L	224.31 ± 69.37	216.71 ± 61.36	230.63 ± 59.41	0.730

^†^*P* < 0.05, compared to normal body mass index patients

*ALB* Albumin, *ALT* Alanine aminotransferase, *AST* Aspartate aminotransferase, *BSA* Body surface area, *DBIL* Direct bilirubin, *GFR* Glomerular filtration rate, *HDL* High-density lipoprotein, *HGB* Hemoglobin, *LAD* Left atrial anteroposterior diameter, *LDL* Low-density lipoprotein, *LVDs* Left ventricular anteroposterior diameter, *LVEF* Left ventricular ejection fraction, *LVFS* Left ventricular shortening fraction, *NYH*A New York Heart Association, *PA* Prealbumin, *PLT* Platelet, *RAD* Right atrial transverse diameter, *RVDs* Right ventricular anteroposterior diameter, *SCr* Serum creatinine, *SPAP* Systolic pulmonary artery pressure, *TBIL* Total bilirubin, *TC* Total cholesterol, *TG* Triglyceride, *UA* Uric acid.

**Table 2:** Type of robotic cardiac surgery

Variable	Normal	Lean	Overweight and 0bese	*P*-value
	*(n* = 81)	(*n* = 17)	(*n* = 48)	
ASD, *n*(%)	37(45.68)	10(58.82)	22(45.83)	0.602
VSD, *n*(%)	7(8.64)	2(11.76)	1(2.08)	0.255
MVR, *n*(%)	11(13.58)	11(64.71)	1(2.08)	0.186
MVP, *n*(%)	7(8.64)	4(23.53)	4(8.33)	0.162
AVR, *n*(%)	1(1.23)	0(0)	3(6.25)	0.187
TVR, *n*(%)	2(2.47)	0(0)	0(0)	0.449
LAM, *n*(%)	12(14.81)	0(0)	5(10.42)	0.215
Concomitant TVP, *n*(%)	9(11.11)	3(17.65)	8(16.67)	0.600
Others, *n*(%)	5(6.17)	0(0)	2(4.17)	0.545

*ASD* Atrial septal defect, *AVR* Aortic valve replacement, *LAM* Left atrial myxoma, *MVP* Mitral valve plasty, *MVR* Mitral valve replacement, *TVP* Tricuspid valve plasty, *TVR* Tricuspid valve replacement, *VSD* Ventricular septal defect, *Others* Endocardial cushion defect, Total anomalous pulmonary venous drainage, Left ventricular tumor.

CSA-AKI: 37 patients (25.34%) had postoperative CSA-AKI, 28 (19.18%) had stage 1 CSA-AKI, 6 (4.11%) had stage 2 CSA-AKI, 3 (2.05%) had stage 3 CSA-AKI, and 1 (0.68%) had renal failure dialysis. (Tables 3 and 4).

**Table 3:** Patient operation and postoperative data

Variable	Normal	Lean	Overweight and 0bese	*P*-value
	(*n* = 81)	(*n* = 17)	(*n* = 48)	
Operative time, min	331.38 ± 116.84	339.24 ± 97.19	381.67 ± 129.92^†^	0.068
CPB time, min	126.19 ± 77.46	138.41 ± 82.35	157.98 ± 86.63^†^	0.102
Cross-clamp time, min	73.69 ± 59.92	85.94 ± 65.58	89.79 ± 65.72	0.344
Intraoperative RBC transfusion ≥ 800ml, *n*(%)	11(13.58)	2(11.76)	4(8.33)	0.673
Intraoperative plasma transfusion ≥ 800ml, *n*(%)	3(3.71)	0(0)	4(8.33)	0.308
Drainage on postoperative day 1, ml	354.88 ± 323.03	356.76 ± 338.55	347.71 ± 294.76	0.991
Tracheal intubation time, h	13.17 ± 7.43	13.85 ± 7.63	14.93 ± 7.43	0.433
Intensive care unit stay, h	52.99 ± 17.33	56.18 ± 29.19	61.43 ± 23.33^†^	0.092
Postoperative hospital stay, day	9.01 ± 2.71	9.76 ± 5.83	10.65 ± 4.67^†^	0.072
Postoperative high-dose transfusion, *n*(%)	12(14.81)	2(11.76)	7(14.58)	0.948
Arrhythmia, *n*(%)	10(12.34)	2(11.76)	11(22.92)	0.254
Pulmonary infection, *n*(%)	25(30.86)	3(17.65)	18(37.50)	0.317
CSA-AKI, n(%)	15(18.52)	3(17.65)	19(39.58)^†^	0.021
Hepatic impairment, *n*(%)	11(13.58)	1(5.88)	12(25.00)	0.111
Abnormal bilirubin metabolism, *n*(%)	51(62.96)	12(70.59)	32(66.67)	0.806
Hypoalbuminaemia, *n*(%)	43(53.08)	11(64.71)	29(60.42)	0.570
Hypoprealbuminaemia, *n*(%)	13(16.05)	6(35.29)	7(14.58)	0.133

^†^*P* < 0.05, compared to normal body mass index patients

*CPB* Cardiopulmonary bypass, *CSA-AKI* Acute kidney injury associated with cardiac surgery, *RBC* Red blood cells.

**Table 4:** One-Way ANOVA of CSA-AKI after robotic cardiac surgery

Variable	CSA-AKI (*n* = 37)	Not CSA-AKI (*n* = 109)	*P*-value
Age, years	47.84 ± 11.53	41.19 ± 13.10	0.007
Male, *n*(%)	21(56.76)	34(31.19)	0.005
Height, cm	161.57 ± 7.79	160.77 ± 7.72	0.589
Weight, kg	61.35 ± 11.89	57.30 ± 11.13	0.062
BMI, kg/m^2^	23.42 ± 3.78	22.13 ± 3.61	0.063
BSA, m^2^	1.62 ± 0.18	1.56 ± 0.17	0.089
Diabetes, *n*(%)	2(5.41)	1(0.91)	0.098
Hypertension, *n*(%)	8(21.62)	10(9.17)	0.047
Smoking, *n*(%)	13(35.13)	15(13.76)	0.004
Alcohol consumption, *n*(%)	15(40.54)	12(11.01)	<0.001
NYHA			
I, *n*(%)	0(0)	2(1.83)	0.412
II, *n*(%)	18(48.65)	77(70.64)	0.019
III, *n*(%)	19(51.35)	30(27.52)	0.007
LVFS, %	36.49 ± 4.59	36.53 ± 4.71	0.959
LVEF, %	66.38 ± 5.21	66.28 ± 6.52	0.937
LAD, mm	37.51 ± 7.32	34.28 ± 6.54	0.013
LVDs, mm	43.78 ± 6.70	42.51 ± 6.97	0.335
RAD, mm	36.95 ± 6.72	37.88 ± 7.91	0.521
RVDs, mm	26.27 ± 6.42	28.11 ± 8.84	0.246
Tricuspid valve gradient, mmHg	29.24 ± 11.22	26.26 ± 12.38	0.197
SPAP, mmHg	37.32 ± 13.17	35.34 ± 15.50	0.486
Operative time ≥ 300 min, n(%)	29(78.38)	47(43.12)	<0.001
CPB time, min	181.01 ± 89.36	123.49 ± 74.08	<0.001
Cross-clamp time, min	112.32 ± 70.27	69.58 ± 56.04	<0.001
TC, mmol/L	3.96 ± 1.09	3.92 ± 0.93	0.861
TG, mmol/L	1.62 ± 1.13	1.15 ± 0.71	0.003
LDL, mmol/L	2.44 ± 0.71	2.37 ± 0.64	0.562
HDL, mmol/L	1.11 ± 0.33	1.26 ± 0.40	0.039
ALT, U/L	24.63 ± 17.04	19.40 ± 11.71	0.040
AST, U/L	23.41 ± 10.31	22.63 ± 7.79	0.632
TBIL, µmol/L	13.76 ± 6.22	14.25 ± 7.22	0.716
DBIL, µmol/L	2.91 ± 2.38	2.77 ± 1.71	0.717
ALB, g/L	41.25 ± 3.82	41.44 ± 4.19	0.804
PA, mg/L	232.66 ± 64.99	224.62 ± 42.93	0.393
SCr, µmol/L	65.79 ± 17.11	60.82 ± 13.28	0.070
GFR, %	142.79 ± 45.02	144.94 ± 34.86	0.764
UA, µmol/L	340.92 ± 76.86	322.29 ± 92.65	0.273
HGB, g/L	136.65 ± 16.47	136.67 ± 25.66	0.996
PLT, 10^9^/L	222.62 ± 56.52	226.47 ± 67.92	0.757

*ALB* Albumin, *ALT* Alanine aminotransferase, *AST* Aspartate aminotransferase, *BMI* Body mass index, *BSA* Body surface area, *CPB* Cardiopulmonary bypass, *DBIL* Direct bilirubin, *GFR* Glomerular filtration rate, *HDL* High-density lipoprotein, *HGB* Hemoglobin, *LAD* Left atrial anteroposterior diameter, *LDL* Low-density lipoprotein, *LVDs* Left ventricular anteroposterior diameter, *LVEF* Left ventricular ejection fraction, *LVFS* Left ventricular shortening fraction, *NYH*A New York Heart Association, *PA* Prealbumin, *PLT* Platelet, *RAD* Right atrial transverse diameter, *RVDs* Right ventricular anteroposterior diameter, *SCr* Serum creatinine, *SPAP* Systolic pulmonary artery pressure, *TBIL* Total bilirubin, *TC* Total cholesterol, *TG* Triglyceride, *UA* Uric acid.

Clinical outcome: 4 cases were transferred to small incision surgery; 1 case of intraoperative tension pneumothorax; 1 case of intraoperative liver bleeding; 1 case of chylothorax; 1 case died in hospital, 1 case had mitral perivalvular fistula, 3 cases had unplanned secondary thoracotomy for hemorrhage, 1 case had cerebral infarction, 1 case had myocardial infarction, and 5 cases had postoperative delirium.

### Comparison of preoperative data

There were statistically significant differences in among age (*P*<0.001), height (*P* = 0.025), weight (*P*<0.001), BSA (*P*<0.001), diabetes(*P* = 0.044), left atrial anteroposterior diameter (LAD) (*P* = 0.022), triglyceride (TG) (*P* = 0.001), and high-density lipoprotein (HDL) (*P* = 0.008) the three groups.(all *P* < 0.05).(Table 1).

### Comparison of surgical and postoperative data

Intraoperative data showed that there was no statistical difference between the lean group and the normal group, and the operation time and CPB time in the overweight and obese groups were significantly higher than those in the normal group (*P* < 0.05). Postoperative clinical outcomes showed that there was no statistical difference between the lean group and the normal group; the intensive care unit stay and postoperative hospital stay in the overweight and obese group were significantly higher than those in the normal group (*P* < 0.05), and the risk of postoperative CSA-AKI was significantly increased (*P* = 0.021). (Table 3).

### Risk factors of CSA-AKI after robotic cardiac surgery

One-Way ANOVA showed significant differences in age (*P* = 0.007), male (*P* = 0.005), hypertension (*P* = 0.047), smoking (*P* = 0.004), alcohol consumption (*P*<0.001), NYHA II (*P* = 0.019) and III (*P* = 0.007), LAD (*P* = 0.013), operative time ≥ 300 min (*P*<0.001), CPB time (*P*<0.001), cross-clamp time (*P*<0.001), TG (*P* = 0.003), HDL (*P* = 0.039), and alanine aminotransferase (ALT) (*P* = 0.040). (Table 4). Multiple Binary Logistic Regression Analysis was performed for factors with univariate *P* < 0.01. Results showed that TG (*OR* 1.772, 95% *CI* 1.068–2.942, *P* = 0.027) and operation time ≥ 300 min (OR 3.823, 95% CI 1.098–13.308, *P* = 0.035) were independent risk factors of CSA-AKI after robotic cardiac surgery. (Table 5).

**Table 5:** Multiple Binary Logistic Regression Analysis of CSA-AKI after robotic cardiac surgery

Variable	*β*	*SE*	*P*-value	*OR*	95% *CI*	
Age	0.030	0.021	0.164	1.030	0.988	1.074
Male	-0.101	0.560	0.857	0.904	0.302	2.708
NYHA	0.287	0.537	0.592	1.333	0.465	3.818
Smoking	0.325	0.766	0.672	1.384	0.308	6.215
Alcohol consumption	1.269	0.714	0.075	3.559	0.879	14.415
TG	0.572	0.259	0.027	1.772	1.068	2.942
Operative time ≥ 300 min	1.341	0.636	0.035	3.823	1.098	13.308
CPB time	-0.007	0.009	0.416	0.993	0.976	1.010
Cross-clamp time	0.010	0.011	0.349	1.010	0.989	1.032
Constant	-6.081	1.960	0.002			

*CPB* Cardiopulmonary bypass, *NYHA* New York Heart Association, *TG* Triglyceride.

## Discussion

Robotic cardiac surgery has the characteristics of small trauma, accurate operation, rapid postoperative recovery, fewer complications, short intensive care unit stay, short hospital stay, greatly improving hospital work efficiency, improving the prognosis of patients and postoperative quality of life [11–13]. Robotic cardiac surgery has been proved to be safe and effective. Robotic cardiac surgery also has drawbacks, requiring longer operating times, significantly higher costs than conventional surgery, and for obese or small thorax patients, the system may not be usable due to the lack of adequate space and vision [14].

Robotic cardiac surgery is particularly important for the selection of patient height and weight, and appropriate surgical patients need to be comprehensively evaluated in combination with imaging before surgery. Obese patients usually have a short thorax, making it extremely difficult to expose the surgical pack, and narrow operating space is more likely to lead to mutual interaction between the robotic arm and assistant devices, device exchange, and delivery difficulties. In addition, robotic cardiac surgery uses left lung one-lung ventilation. Obese patients usually have poor postoperative cough and expectoration ability, resulting in increased incidence of postoperative atelectasis and pulmonary infection and prolonged intensive care unit stay. This study did not find increased incidence of respiratory complications in overweight and obese patients, but found significantly prolonged intensive care unit stay and postoperative hospital stay. Obese patients have been shown to initiate and worsen conditions such as type 2 diabetes, obstructive sleep apnea, and kidney disease, which have poor preoperative conditions and comorbidities and may influence the choice of the final surgical strategy.

Current studies have shown that more than 37% of patients undergoing cardiac surgery are obese [15], therefore, robotic cardiac surgery in obese patients has many difficulties. Obesity is generally considered to be an important risk factor for cardiovascular mortality, leading to increased morbidity, mortality, and health care costs [1, 2], but studies have found that obesity is associated with reduced mortality after cardiac surgery, and it is believed that obese patients seem to have a better short-term and long-term prognosis than lean patients, that is the obesity paradox, and such patients are mostly coronary artery bypass grafting [3, 4]. In recent years, several studies have suggested that obese patients may be at no different risk for cardiac surgery than non-obese patients, particularly in terms of mortality [5–7]. In this study, we found that overweight and obese patients had a significantly increased risk of CSA-AKI after surgery, and significantly prolonged postoperative intensive care unit stay and hospital stay, contrary to the cardio-obesity paradox, consistent with previous studies [16, 17]. This study has the limitation of small sample and lack of follow-up of mid- and long-term outcomes after surgery, which cannot completely reflect the obesity paradox after robotic cardiac surgery and is expected to be confirmed by subsequent studies.

Acute kidney injury (AKI) is the most common complication after cardiac surgery. According to statistics, the incidence of CSA-AKI varies from 5–42% [18]. In this study, we found that the incidence of CSA-AKI after robotic cardiac surgery was 25.34%, which is consistent with previous reports. CSA-AKI is an important cause of in-hospital mortality and is second only to sepsis in intensive care units and is independently associated with increased morbidity and mortality [19]. Severe CSA-AKI is independently associated with a 3 to 8 fold increase in perioperative mortality, prolonged intensive care unit and hospital stays, and increased costs of care [20]. Several major injury pathways may be involved in the development of CSA-AKI, including hypoperfusion, ischemia-reperfusion injury, neurohumoral activation, inflammation, oxidative stress, nephrotoxins, and mechanical factors. All these routes of injury can occur preoperatively, intraoperatively, and postoperatively. Adverse outcomes in obese cardiac surgery patients are associated with altered branched-chain amino acid catabolism in adipose and cardiac tissue [21]. Obese patients exhibit increased oxidative stress, endothelial dysfunction, and inflammation [22]. Obesity can significantly alter renal hemodynamics, which may explain the increased susceptibility to AKI in obese patients. In addition, obese patients often develop pulmonary heart disease due to hypoventilation, sleep apnea, and pulmonary hypertension, resulting in sodium dependence and peripheral venous congestion, which in turn leads to increased renal venous pressure, thereby reducing urine formation.

In this study, we found that preoperative TG was an independent risk factor for postoperative CSA-AKI. Overweight and obese patients had significantly increased TG levels and significantly decreased HDL levels. HDL had systemic anti-inflammatory and antioxidant properties. Preoperative high HDL levels were associated with decreased CSA-AKI after cardiac surgery. Preoperative and perioperative statin therapy enhanced this association [23]. However, scholars currently have contradictions about the role of preoperative statins in the prevention of CSA-AKI [24, 25]. This study suggests that preoperative TG levels are independently associated with CSA-AKI after robotic cardiac surgery and is a novel finding that awaits confirmation by subsequent studies. Surgery itself may be associated with an increased risk of CSA-AKI, and it has been found that prolonged CPB time, prolonged aortic cross-clamp time, non-pulsatile flow on CPB, hemolysis, and hemodialysis may increase the risk of CSA-AKI [26]. In this study, we found that operation time ≥ 300 min was independently associated with postoperative CSA-AKI, and prolonged operation time indicated that the more difficult the operation, the increased incidence of postoperative complications.

## Conclusion

Overweight and obese patients had significantly prolonged intensive care unit stay and postoperative hospital stay after robotic cardiac surgery, and the incidence of postoperative CSA-AKI was significantly increased, which did not support the obesity paradox; further analysis showed that postoperative CSA-AKI was independently associated with preoperative TG and operation time ≥ 300 min. This study is a small single-center retrospective study, lacking postoperative medium- and long-term follow-up data, and is expected to be confirmed by subsequent studies.

## Electronic supplementary material

Below is the link to the electronic supplementary material.

Additional File: Clinically collected data

## Data Availability

The datasets used and/or analysed during the current study are available from the corresponding author on reasonable request.

## Abbreviations

AKI: Acute kidney injury
ALB: Albumin
ALT: Alanine aminotransferase
ASD: Atrial septal defect
AST: Aspartate aminotransferase
AVR: Aortic valve replacement
BMI: Body mass index
BSA: Body surface area
CPB: Cardiopulmonary bypass
CRRT: Continuous renal replacement therapy
CSA-AKI: Acute kidney injury associated with cardiac surgery
CT: Computed tomography
DBIL: Direct bilirubin
GFR: Glomerular filtration rate
HDL: High-density lipoprotein
HGB: Hemoglobin
LAD: Left atrial anteroposterior diameter
LAM: Left atrial myxoma
LDL: Low-density lipoprotein
LVDs: Left ventricular anteroposterior diameter
LVEF: Left ventricular ejection fraction
LVFS: Left ventricular shortening fraction
MVP: Mitral valve plasty
MVR: Mitral valve replacement
NYHA: New York Heart Association
PA: Prealbumin
PLT: Platelet
RAD: Right atrial transverse diameter
RBC: Red blood cells
RVDs: Right ventricular anteroposterior diameter
SCr: Serum creatinine
SD: Standard deviation
SPAP: Systolic pulmonary artery pressure
TBIL: Total bilirubin
TC: Total cholesterol
TEE: Transesophageal echocardiography
TG: Triglyceride
TTE: Transthoracic echocardiography
TVP: Tricuspid valve plasty
TVR: Tricuspid valve replacement
UA: Uric acid
VSD: Ventricular septal defect
